# Odontological Society of Pennsylvania

**Published:** 1890-01

**Authors:** 


### ODONTOLOGICAL SOCIETY OF PENNSYLVANIA.

The Odontological Society of Pennsylvania held its regular monthly meeting in Justi's Hall, November 2, 1889. A paper was read by Dr. Ernest Laplace on fermentation.

### DISCUSSION ON PROFESSOR LAPLACE'S PAPER.

*Dr. Sudduth.*—I would like Dr. Laplace to answer a few questions which will bring out points which may not be as clear in the minds of some as they should be. First, I would like to know what effect the "alteration of the soil" has to prevent the growth of these germs?

*Dr. Laplace.*—I can only say that it is a fact which has been observed from the time Jenner inoculated cow-pox against small-pox; but just why this should render the soil unfit for their growth I am unable to say.

*Dr. Sudduth.*—Might there not be a slight change in the albuminoid substances of the body?

*Dr. Head.*—Might not the weaker microbe act on the stronger as a restraint?

*Dr. Laplace.*—As an illustration, suppose two young men, brothers, both in health, are exposed to the same infection. One from exposure contracts pneumonia. After the one has recovered from the pneumonia they are again exposed, this time, however, to the germs of tuberculosis. The germs which before fell harmless on the soil of each of their lungs now find suitable soil in the lungs of the one who has had pneumonia, and take root and grow, while they are still harmless to the one who has never had pneumonia.

I could entertain you a good while with many of the experiments of Pasteur. But as this one illustrates the point in question I will relate it. Pasteur was told that a chicken could not be inoculated with anthrax or splenic fever. In experimenting with the cultures of the anthrax bacillus he found that, when grown, at a temperature of 42° C., these germs were not virulent; but when cultivated at 37° C., they were highly virulent to its growth. He then took the temperature of a chicken and found it to be 42°. He then went down to the Academy of Science, and took his chicken and a basin of ice-water. He plunged the chicken into the ice-water until its temperature was reduced to 37°, and then inoculated it. In twenty-four hours the chicken was dead from anthrax.

*Dr. Kirk.*—Suppose you have treated a tooth thus theoretically antiseptically, and some time—six months or more afterwards—there develops in that tooth an alveolar abscess, then would you say that there had been left there some germs, or, that the germs had since been introduced?

*Dr. Laplace.*—We know that germs will travel through mucous membrane through the stomata or little mouths of blood-vessels. They may get there in this way or perhaps be carried direct by the blood-vessels, in some diseased state of the blood, such as erysipelas or pyæmia.

*Dr. Faught.*—Why does it select this particular tooth and not another?

*Dr. Laplace.*—I can only give you the reason which I gave before, that of lowered vitality. To have a correct idea of the process, one must take into consideration the histology of the part. When a part of the body is irritated by the germs, more blood is sent to that part, and the white blood-corpuscles pass through the vessel walls and attack the microbes, and, if in sufficient numbers and strength, destroy them, and the part regains its health. If, however, the germs are in too great numbers, they will gain the upper hand in the deadly strife, and disease will follow. A Russian pathologist, in looking at this conflict, discovered the microbe inside the white blood-corpuscle, and from this fact named them *phagocytes*,—the Greek to eat. When a great number of white blood-corpuscles have been thus destroyed by the action of a local irritant, chemical or a living micro-organism, and are retained in that special locality in a certain amount of fluid, we have the pathological product commonly known as *pus.*

*Dr. Sudduth.*—Will the doctor give us some idea of the best antiseptic, and how used?

*Dr. Laplace.*—The first antiseptic used was carbolic acid. This was discarded in favor of the bichloride of mercury, which Koch, in 1882, found to be destructive to the life of the anthrax bacillus in the proportion of 1–1000,—that is, in a watery solution of this strength the germs would be destroyed. But when the mercury was brought in contact with the albumen of the tissue of the bodies, there was thrown down a heavy precipitate of the albuminate of mercury, which rendered the solution inactive. When in Koch's laboratory, taking a subject for special investigation, I undertook to find some way to prevent this deposit and keep the solution active. I took this deposit and planted therein the germs of suppuration, and they grew and thrived, thus proving the uselessness of it where this deposit was. After a long series of experiments, I found that by adding five per cent. of acid to the solution, I had not only solved the question of preventing the deposit, but had rendered the solution much stronger and efficacious.

*Dr. James Truman.*—From the time when Senwenhoeck, in 1680, found that yeast consisted of globular particles to the period when Schwan and Latour independently proved them to be vegetable cells, was a period of less activity than that which followed; yet it was the commencement of that greater work that has been so prolific in results in more recent times.

The views of Schwan naturally led to opposition, and Leibig's molecular action theory held its place as the most prominent, and, at the same time, most difficult theory to combat. When Schroeder proved that he could filter out the spores from the air, another advance was made, and rendered possible other steps in the investigation of this subject. Bastian aided materially in this labor; but his spontaneous generation theory has not found a place, and he failed to sustain the views of Leibig that the molecular movements were the cause of fermentation by adding on his own, that they actually became vital. From this period to that more recent, when Koch proved the existence of the tubercle bacillus, there has been continued progress, and the germ theory of disease has ceased to be a speculation. The theories brought forward from time to time, to combat it have fallen into deserved oblivion.

The address and the remarks of Professor Laplace this evening abundantly prove the thoroughness with which the whole subject of fermentation has been treated by those to whom we are most indebted for its elucidation. To us, as dentists, this subject appeals with daily increasing force. We are constantly brought in contact with conditions that must be intelligently met, and it is only by a satisfactory knowledge of fermentation and antisepsis that these can be properly met. To Professor Miller we owe a debt of gratitude that he has, unquestionably, solved the problem of dental caries, and his labors in investigating the ferments of the oral cavity have shown the necessity for an equal degree of thoroughness in our operations with that which made Lister's name famous in those of general surgery.

When it is fully recognized that the mouth is, without doubt, the culture place for many pathogenic forms, and that many of the zymotic diseases have their genesis therein, more attention will be paid to its condition by general practitioners than is given at present. When the dentists of to-day appreciate the possibilities of fermentation, they will govern all their operations in the mouth with a care as to possible results from infection. The time is certainly not far off when prophylactic measures will be studied with special reference to the mouth and its secretions. Efforts will be made to destroy these germs as they enter this vestibule of the entire organism, and prevent them from beginning their insidious and poisonous work. That this is to be the labor of the future I have no doubt. That it will, in degree, stamp out many forms of disease I am equally well satisfied, and the time will, in my opinion, most assuredly come when we will know less of the destructive work of the tubercle bacillus than we now do, but this will be accomplished in advance of pathological conditions.

*Dr. Sudduth.*—I would say for the benefit of those who are not aware of the fact that tablets of the acid sublimate, put up after Dr. Laplace's formula, are to be had at Mulford's drug store.

